# Clinicopathologic implications of the miR-197/PD-L1 axis in oral squamous cell carcinoma

**DOI:** 10.18632/oncotarget.19842

**Published:** 2017-08-03

**Authors:** Hyein Ahn, Jeong Mi Yang, Hyojin Kim, Jin-Haeng Chung, Soon-Hyun Ahn, Woo-Jin Jeong, Jin Ho Paik

**Affiliations:** ^1^ Department of Pathology, Seoul National University Bundang Hospital, Seoul National University College of Medicine, Seongnam, Korea; ^2^ Department of Otorhinolaryngology, Seoul National University Bundang Hospital, Seoul National University College of Medicine, Seongnam, Korea

**Keywords:** PD-L1, oral squamous cell carcinoma, miR-197, microRNA, tumor-infiltrating lymphocytes

## Abstract

Immune escape of a tumor from tumor-infiltrating lymphocytes (TILs) is induced by PD-L1, which is suppressed by miR-197. We investigated the clinicopathologic implications of the miR-197/PD-L1 axis and its effects on TILs and the clinicopathologic features of oral squamous cell carcinoma (OSCC). We used RT-PCR and immunohistochemistry in 68 OSCC patients to analyze the correlations between tumoral expression of miR-197 and PD-L1 and the degree of tumoral invasion by TILs (CD3+, CD4+, CD8+, PD-1+, FoxP3+, and CD20+ lymphocytes). PD-L1 levels correlated inversely with miR-197 but correlated positively with TILs. The aggressive features of OSCC, including high stage, angiolymphatic invasion, perineural invasion, and death, were associated with TIL depletion. High T stage (T4) tumors also had low PD-L1 but had high miR-197 expression. In a univariate survival analysis of the full cohort, high miR-197 was associated with poor overall survival, whereas high PD-L1 expression (2+) associated with good overall survival. In a multivariate analysis stratified based on miR-197 (median), high PD-L1 expression (2+) was an independent favorable prognostic factor for overall survival (*P* = 0.040) in the miR-197^high^ subgroup but not the miR-197^low^ subgroup. These findings may have clinicopathologic implications for the miR-197/PD-L1 axis and TILs in OSCC.

## INTRODUCTION

Oral cancer accounts for 1% to 5% of human malignancies, and its occurrence has increased for more than a decade [1]. Oral squamous cell carcinoma (OSCC) is the predominant type and comprises approximately 90% of all oral cancers [2]. OSCC occurs mostly in males with long-term exposure to tobacco and alcohol who are in their fifth to seventh decade of life [3]. The prognosis of OSCC remains poor because of the tendency of the cancer to metastasize [4]. Despite recent advances in combined surgery, chemotherapy, and radiotherapy, the survival rates of patients with OSCC has shown no improvement over the past few decades [2]. Therefore, an understanding of the molecular mechanism underlying progression of OSCC in the context of the tumor microenvironment is needed for improvement of the therapeutic strategies for the disease.

Programmed cell death ligand-1 (PD-L1) is a cell-surface glycoprotein that induces T cell anergy and apoptosis by activating the PD-1 receptors on T lymphocytes [5]. Normally, PD-L1 is a factor in maintaining immunologic homeostasis, but in many cancers PD-L1 is overexpressed on tumor cells as well as on subsets of immune cells, including T cells, B cells, macrophages, and dendritic cells. Blocking the PD-L1/PD-1 pathway reverses the immune escape of the tumor and improves the anticancer immune responses in microenvironments containing tumor-infiltrating lymphocytes (TILs) [6]. PD-L1 has gained attention for its benefits in clinical trials of cancer immunotherapy. However, the number of studies on the biologic mechanisms and effects of PD-L1 in OSCC is limited [7, 8], and the clinicopathologic implications, including prognostic values and associations with TILs, have not been clarified.

MicroRNAs (miRNAs) are single-stranded, non-coding short RNAs consisting of 20 to 22 nucleotides that inhibit the translation of genes by disrupting specific messenger RNAs. In recent studies, the expression profiles of miRNAs in OSCC were examined and some miRNAs were found to correlate with tumorigenesis and cancer progression [9, 10]. MiR-197 is associated with a broad range of pathologic conditions, including malignancies and various non-neoplastic diseases [11]. Studies suggest an miR-197-induced regulating mechanism of PD-L1 in tumor cells via the miR-197/CKS1B/STAT3 pathway in non-small cell lung cancer (NSCLC) [12, 13]. In those studies, downregulation of miR-197 was suggested as the promoter of chemoresistance, having therapeutic potential by miR-197 replacement, particularly in PD-L1-positive NSCLC patients.

In addition to miR-197, PD-L1 is regulated by other microRNAs, including miR-138-5p, miR-513, miR-570, miR-34a, and miR-200, the functions of which have not yet been elucidated in OSCC [14-18]. Considering previously reported clinically relevant signaling pathways involving CKS1B and STAT3 in OSCC and the known miR-197/CKS1B/STAT3/PD-L1 pathway [12, 13, 19-21], we focused on the miR-197/PD-L1 axis and TILs.

In the present study, we contribute to a better understanding of the clinicopathologic implication of the miR-197/PD-L1 axis and TILs in OSCC. We analyzed the expression of miR-197 and PD-L1 and analyzed the number of recruited TILs (CD3+, CD4+, CD8+, PD-1+, FoxP3+, and CD20+ lymphocytes) and the correlations with various clinicopathologic features and prognosis in OSCC patients.

## RESULTS

### Clinicopathologic characteristics of OSCC patients

The various clinicopathologic features of OSCC patients are summarized in Table 1. They were from 45 male patients and 23 female patients, with a median age of 57.7 years (range = 23-84 years). The distribution according to pathologic TNM (pTNM) staging classification by the seventh edition of the AJCC was as follows: 18 patients (26.5%) were I; 17 patients (25.0%) were II; 9 patients (13.2%) were III, and 24 patients (35.3%) were IV. Among the 68 patients, grading categories of immunohistochemical intensity of PD-L1 expression in tumor cells were distributed as no staining (0; 23/68, 33.8%), weak positivity (1+; 23/68, 33.8%), and moderate to strong positivity (2+; 22/68, 32.4%). The numbers of CD3+, CD4+, CD8+, PD-1+, FoxP3+, and CD20+ TILs are shown in Table 1. PD-1+ TILs and FoxP3+ TILs were less dominant, compared with CD3+, CD4+, CD8+, and CD20+ TILs.

**Table 1:** Clinicopathologic features of oral squamous cell carcinoma (n = 68)

Variables	
Age, y	
mean (range)	57.7 (23-84)
Sex	
F	23 (33.8%)
M	45 (66.2%)
Angiolymphatic invasion	
Absent	49 (72.1%)
Present	19 (27.9%)
Perineural invasion	
Absent	49 (72.1%)
Present	19 (27.9%)
Tumor size (largest dimension, mm)	28.9 (12-85)
pT	
pT1	21 (30.9%)
pT2	26 (38.2%)
pT3	4 (5.9%)
pT4a	17 (25.0%)
pN	
pN0	42 (61.8%)
pN1	15 (22.1%)
pN2b	10 (14.7%)
pN2c	1 (1.5%)
pTNM	
I	18 (26.5%)
II	17 (25.0%)
III	9 (13.2%)
IV	24 (35.3%)
Number of TILs	
CD3+ TILs, mean±SD	42.5±37.0
CD4+ TILs, mean±SD	32.6±26.7
CD8+ TILs, mean±SD	27.5±22.2
PD-1+ TILs, mean±SD	6.8±6.9
FoxP3+ TILs, mean±SD	9.4±9.4
CD20+ TILs, mean±SD	21.7±23.2
PD-L1 expression	
0	23 (33.8%)
1+	23 (33.8%)
2+	22 (32.4%)
Survival	
Alive	45 (66.2%)
Death	23 (33.8%)
Relapse	
No relapse	49 (72.1%)
Relapse	19 (27.9%)
Neoadjuvant treatment	
Not done	65 (95.6%)
Done	3 (4.4%)
Additional treatment^**^	
Not done	31 (45.6%)
RTx only	26 (38.2%)
CTx only	2 (3.0%)
CCRT or SCRT	9 (13.2%)

TILs, tumor-infiltrating lymphocytes; SD, standard deviation; CCRT, concurrent chemoradiotherapy; SCRT, sequential chemoradiotherapy; CTx, chemotherapy; RTx, radiotherapy.

*Neoadjuvant treatment (3 cases) includes one neoadjuvant CCRT, one neoadjuvant RTx, and one neoadjuvant CTx.

**Additional treatment (37 cases) includes neoadjuvant treatment (3/37, 8%) and postoperative therapy (34/37, 92%).

### Associations among clinicopathologic features and miR-197, PD-L1, and TILs in OSCC patients

**Figure 1:**
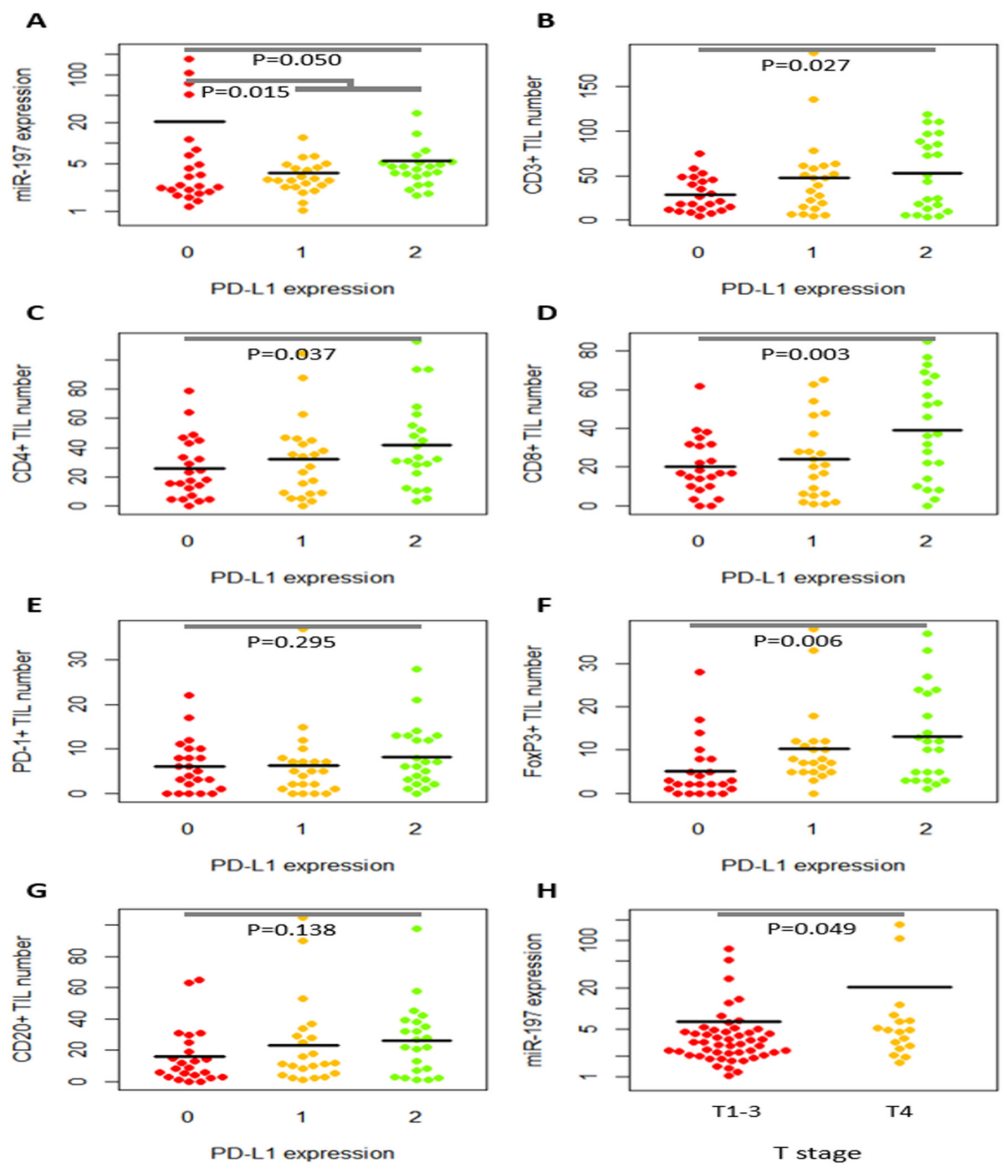
Dot plots of relative miR-197 expression level and tumor-infiltrating lymphocytes (TILs) according to PD-L1 expression and T stages MiR-197 expression level **(A)** and the numbers of CD3+ **(B)**, CD4+ **(C)**, CD8+ **(D)**, PD-1+ **(E)**, FoxP3+ **(F)**, and CD20+ **(G)** tumor-infiltrating lymphocytes (TILs) were illustrated according to PD-L1 level. PD-L1 expression is inversely correlated with miR-197 level (A) but tended to have a positive correlation with TILs (B–G). MiR-197 level is higher in T4 disease than in T1-T3 disease **(H)**.

Clinicopathologic features were not significantly different between miR-197^low^ (<median) and miR-197^high^ (>median) subgroups or between low PD-L1 (0/1+) and high PD-L1 (2+) subgroups (Supplementary Tables 1 and 2). In the quantitative analysis of miR-197 and TILs according to clinicopathologic features (Table 2), miR-197 level was higher in male patients (*P* = 0.049), and PD-1+ TILs were increased in older patients (*P* = 0.018). The parameters of tumor aggressiveness were associated with depletion of various TILs. Decreased PD-1+, FoxP3+, and CD20+ TILs were associated with higher AJCC stages (3-4) (*P* = 0.010, *P* = 0.004, and *P* = 0.035, respectively). Specifically, high T stage (T3-T4) was correlated with low numbers of CD3+, CD8+, PD-1+, FoxP3+, and CD20+ TILs (*P* = 0.019, *P* = 0.030, *P* = 0.019, *P* = 0.005, and *P* = 0.004, respectively), and high N stage (N1-N2) disease had fewer PD-1+ TILs in the primary tumor (*P* = 0.020). In addition, the tumors with angiolymphatic invasion had fewer CD20+ TILs (*P* = 0.006), and perineural invasion was associated with fewer PD-1+ and FoxP3+ TILs (*P* = 0.022 and *P* = 0.017). CD4+ TIL numbers were lower in patients who died (*P* = 0.035). When T stage was divided into T1-T3 versus T4, T4 disease had a higher miR-197 level (*P* = 0.049; Figure 1 and Table 3) but low PD-L1 expression (*P* = 0.002; Table 3). Taken together, aggressive features were associated with higher miR-197 level, but depleted TILs and low PD-L1 expression.

**Table 2:** Average numbers of tumor-infiltrating lymphocytes and level of miR-197 according to clinicopathologic features of oral squamous cell carcinoma (n = 68)

Clinicopathologic variables	Number of patients (total n=68)	CD3+		CD4+		CD8+		PD-1+		FoxP3+		CD20+		miR-197	
		Mean ± SD	*P*-value	Mean ± SD	*P*-value	Mean ± SD	*P*-value	Mean ± SD	*P*-value	Mean ± SD	*P*-value	Mean ± SD	*P*-value	Mean ± SD	*P*-value
Age			0.579		0.709		0.725		**0.018**		0.395		0.569		0.282
<55	25	39.1±40.8		31.0±24.6		26.2±20.7		4.6±3.8		8.1±7.5		19.6±22.6		5.4±9.9	
≥55	43	44.6±35.0		33.5±28.1		28.2±23.3		8.1±7.9		10.2±10.4		23.0±23.8		12.4±31.2	
Gender			0.512		0.494		0.434		0.706		0.175		0.466		**0.049**
Male	45	40.3±33.4		34.2±28.4		26.0±20.8		7.0±7.0		10.4±10.8		23.2±23.5		13.0±31.1	
Female	23	46.7±43.6		29.5±23.3		30.5±25.0		6.4±6.8		7.6±5.9		18.8±22.9		3.6±2.3	
AJCC Stage			0.057		0.145		0.116		**0.010**		**0.004**		**0.035**		0.538
Stage 1-2	35	50.7±35.0		37.2±30.9		31.6±24.2		8.8±8.4		12.5±10.2		27.4±23.3		8.0±14.6	
Stage 3-4	33	33.5±37.6		27.7±20.7		23.1±19.3		4.6±3.8		6.0±7.3		15.5±21.9		11.8±33.8	
AJCC Tumor Stage			**0.019**		0.097		**0.030**		**0.019**		**0.005**		**0.004**		0.280
pT1-2	47	49.6±39.2		36.2±28.7		30.9±23.9		7.8±7.8		11.4±9.5		26.1±25.6		6.6±12.8	
pT3-4	21	26.9±26.3		24.6±19.7		19.9±16.0		4.5±3.5		5.0±7.7		12.1±12.7		17.0±41.9	
AJCC Lymph node Stage			0.454		0.422		0.739		**0.020**		0.128		0.343		0.982
pN0	42	45.1±34.7		34.7±29.5		28.2±23.7		8.1±8.0		10.8±10.1		23.8±22.8		9.9±20.3	
pN1-2	26	38.1±40.9		29.3±21.4		26.4±20.0		4.7±3.8		7.1±8.0		18.2±24.1		9.8±33.0	
Angiolymphatic invasion			0.286		0.437		0.163		0.197		0.465		**0.006**		0.336
Not identified	49	45.5±39.3		32.0±27.3		27.9±22.3		8.0±7.5		9.9±9.2		25.3±25.3		8.3±18.8	
Present	19	34.5±29.5		34.1±25.6		26.5±22.8		3.7±3.7		8.0±10.2		12.1±12.3		13.7±38.6	
Perineural invasion			0.947		0.783		0.815		**0.022**		**0.017**		0.455		0.439
Not identified	49	42.3±30.7		32.0±27.3		27.9±22.3		8.0±7.5		10.8±10.2		23.1±22.0		8.3±18.8	
Present	19	43.1±50.1		34.1±25.6		26.5±22.8		3.7±3.7		5.8±6.2		18.3±26.5		13.7±38.6	
Survival			0.144		**0.035**		0.137		0.459		0.398				0.123
Alive	45	47.3±40.7		37.4±29.2		30.4±21.7		6.3±5.4		10.1±9.4		24.6±25.7		5.1±8.2	
Death	23	33.4±27.1		23.1±17.9		21.9±22.6		7.8±9.2		8.0±9.6		16.3±16.70.170		19.1±41.6	
Relapse			0.923		0.327		0.833		0.612		0.511		0.491		0.333
No relapse	49	42.2±39.2		34.6±27.7		27.9±22.0		7.1±6.7		8.8±8.3		23.0±25.3		7.2±16.4	
Relapse	19	43.2±31.9		27.5±23.8		26.6±23.4		6.1±7.5		10.8±12.1		18.6±17.0		16.7±40.8	
Neoadjuvant treatment			0.109		0.557		0.142		0.477		0.232		0.155		0.751
Not done	65	44.1±37.1		33.0±27.0		28.4±22.4		6.9±7.0		9.7±9.6		22.6±23.4		10.0±26.2	
Done	3	9.0±5.3		23.7±20.4		9.0±6.6		4.0±3.6		3.0±2.0		3.0±2.6		5.2±5.2	
Additional treatment			0.978		0.981		0.996		0.303		0.051		0.634		0.132
Not done	31	42.4±34.7		32.5±28.2		27.5±22.6		7.7±7.6		11.9±10.3		23.2±24.1		5.0±8.7	
Done	37	42.6±39.3		32.7±25.7		27.5±22.3		6.0±6.2		7.4±8.3		20.5±22.8		13.8±33.5	

**Table 3:** Correlations among tumor PD-L1 expression, miR-197 level, tumor-infiltrating lymphocytes, and pathologic stages in oral squamous cell carcinoma (n = 68)

	PD-L1 expression (0 vs. 1+/2+)	CD3+ TIL number	CD4+ TIL number	CD8+ TIL number	PD-1+ TIL number	FoxP3+ TIL number	CD20+ TIL number	T stage (T1-3 vs. T4)	N stage (N0 vs. N1-2)
miR-197 expression	***P* = 0.015** r = −0.293	*P* = 0.316 r = −0.124	*P* = 0.291 r = −0.130	*P* = 0.291 r = −0.130	*P* = 0.787 r = −0.033	*P* = 0.307 r= −0.127	*P* = 0.207 r= −0.156	***P =* 0.049** r = 0.239	*P =* 0.982 r = −0.003
PD-L1 expression (0, 1+, 2+)		***P* = 0.027** r = 0.271	***P* = 0.037** r = 0.254	***P* = 0.003** r = 0.352	*P* = 0.295 r = 0.129	***P* = 0.006** r = 0.333	*P* = 0.138 r = 0.183	*P* = 0.002* r = −0.371*	*P* = 0.648* r = −0.057*
CD3+ TIL number			***P* <0.001** r = 0.585	***P* <0.001** r = 0.659	*P* = 0.169 r = 0.170	***P* <0.001** r = 0.440	***P* <0.001** r = 0.741	***P* = 0.047** r = 0.243	*P* = 0.454 r =−0.093
CD4+ TIL number				***P* <0.001** r = 0.679	*P* = 0.737 r = 0.041	***P* = 0.001** r = 0.386	***P* <0.001** r = 0.420	*P* = 0.225 r = −0.149	*P* = 0.422 r = −0.099
CD8+ TIL number					*P* = 0.155 r = 0.174	***P* <0.001** r = 0.471	***P* <0.001** r = 0.419	*P* = 0.061 r = −0.228	*P* = 0.739 r = −0.041
PD-1+ TIL number						*P* = 0.055 r = 0.235	*P* = 0.115 r = 0.194	*P* = 0.118 r = −0.191	***P* = 0.020** r = −0.246
FoxP3+ TIL number							***P* = 0.017** r = −0.292	***P* <0.001** r = −0.417	*P* = 0.128 r = −0.188
CD20+ TIL number								***P* = 0.029** r = −0.266	*P* = 0.343 r = −0.118
T stage (T1-3 vs. T4)									*P* = 0.044* r = 0.245*

TIL, tumor-infiltrating lymphocyte. *P* and r indicate *P*-value and correlation coefficient from Pearson correlation test.

Asterisk (*) indicates *P*-value and correlation coefficient from Spearman’s rho test.

### Associations between the miR-197/PD-L1 axis and microenvironmental TILs

**Figure 2:**
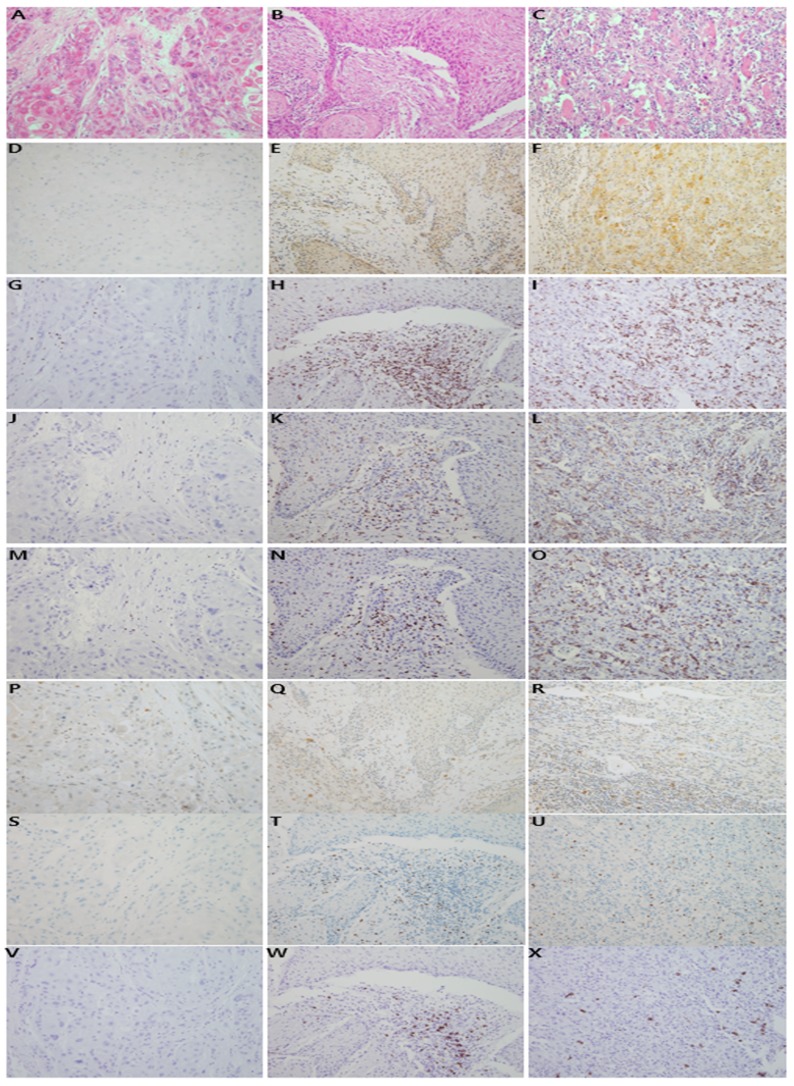
Representative features of histologic and immunohistochemical analyses Histologic features of invasive oral squamous cell carcinomas are shown (hematoxylin and eosin stain, x200; **A, B, C**). Immunostaining of PD-L1 revealed no staining (0; **D**), weak positivity (1+; **E**), and moderate to strong positivity (2+; **F**). Tumor-infiltrating lymphocytes tended to be variably correlated with PD-L1 expression. The infiltrating lymphoid cells were immunostained with CD3 **(G, H, I)**, CD4 **(J, K, L)**, CD8 **(M, N, O)**, PD-1 **(P, Q, R)**, FoxP3 **(S, T, U)**, and CD20 **(V, W, X)**.

Based on research that suggests miR-197 indirectly suppresses PD-L1 expression [12], we analyzed the relation between level of miR-197 and PD-L1. As shown in Figure 1, Figure 2 and Table 3, miR-197 expression is inversely correlated with PD-L1 expression (r = −0.239, *P* = 0.050, for 0, 1+, and 2+ of PD-L1 intensity; r = −0.293, *P* = 0.015, for 0 vs. 1+/2+ of PD-L1 intensity). Because PD-L1 is associated with proliferation and function of TILs, we comparatively analyzed the relations among CD3+, CD4+, CD8+, PD-1+, FoxP3+, CD20+ TILs, and PD-L1 expression (Table 3). As shown in Figure 1 and Table 3, PD-L1 expression in tumor cells was positively correlated with the numbers of CD3+, CD4+, CD8+, and FoxP3+ TILs (*P* = 0.027, *P* = 0.037, *P* = 0.003, and *P* = 0.006, respectively), but not with PD-1+ TILs or CD20+ TILs (*P* > 0.05). When the relation among various TILs was analyzed, frequent positive correlations were observed among CD3+, CD4+, CD8+, FoxP3+, and CD20+ TILs.

### The effects of miR-197 and PD-L1 expression and TILs on prognosis in OSCC

**Figure 3:**
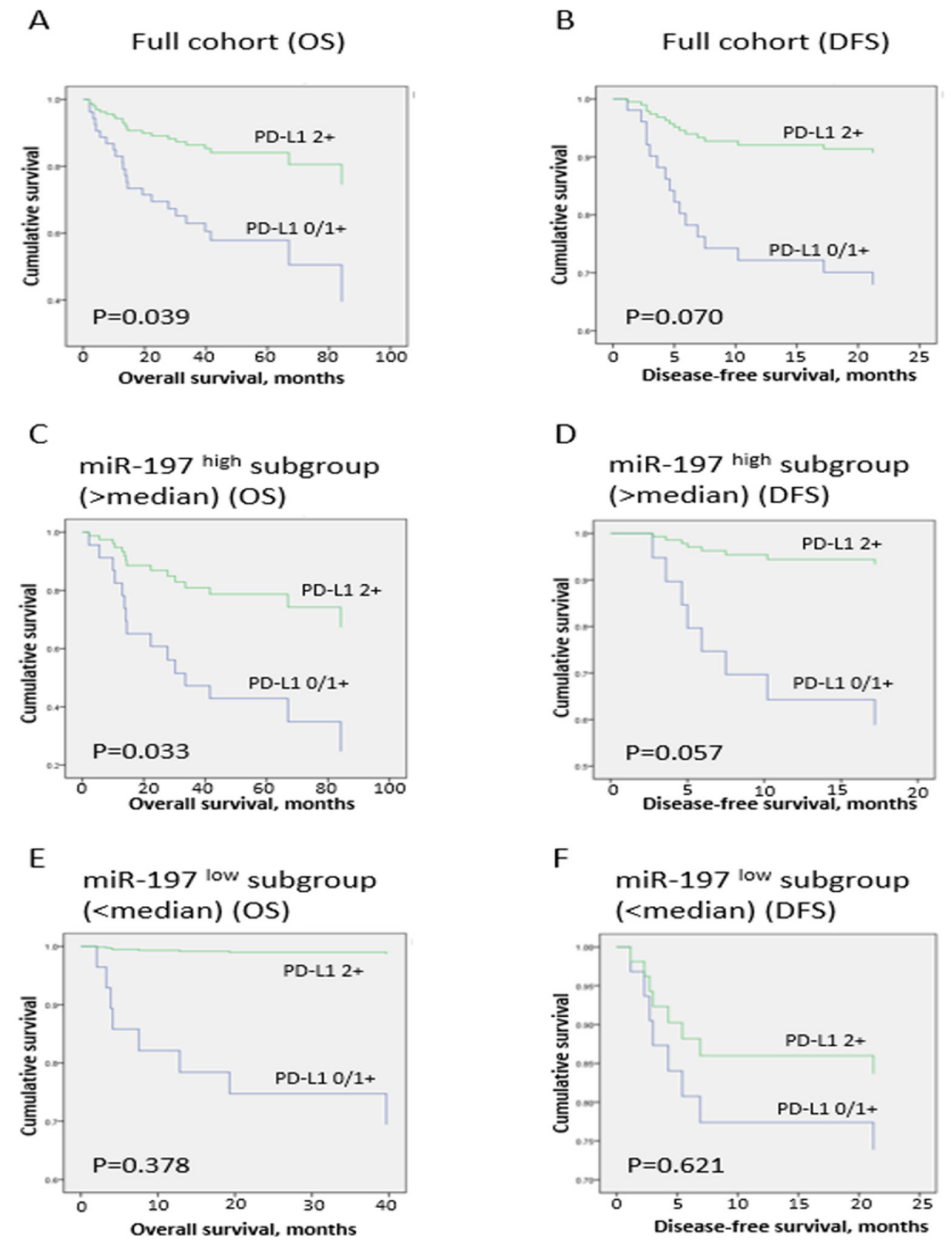
Survival analysis in full cohort and miR-197^high^ and miR-197^low^ subgroups Survival curves for overall survival **(A, C, E)** and disease-free survival **(B, D, F)** in full cohort **(A** and **B)**, miR-197^high^ (>median; **C** and **D**), and miR-197^low^ subgroups (<median; **E** and **F**) in oral squamous cell carcinoma patients.

The association between conventional clinicopathologic factors and prognosis in OSCC patients was evaluated (Table 4 and Figure 3). Univariate analysis revealed that T stage (T3-T4; *P* < 0.001) and angiolymphatic invasion (*P* = 0.001) were predictors of poor OS in OSCC patients. Similarly, T stage (T3-T4; *P* < 0.001), angiolymphatic invasion (*P* = 0.001), and N stage (*P* = 0.007) were associated with poor DFS. High expression of miR-197 was weakly associated with poor OS (*P* = 0.033), and high PD-L1 expression in tumor cells (2+) predicted better OS (*P* = 0.039; Figure 3), as expected from the inverse correlation between miR-197 and PD-L1. In multivariate analysis, T stage (T3-T4) and angiolymphatic invasion were independent poor prognostic factors for OS (*P* < 0.001 and *P* = 0.002), and DFS (*P* = 0.001 and *P* = 0.003), but miR-197 or PD-L1 was not associated with OS or DFS.

**Table 4:** Prognostic factors for overall survival and disease-free survival in oral squamous cell carcinoma (n = 68)

Variables	Univariate analysis		Multivariate analysis	
	HR (95% CI)	*P*-value	HR (95% CI)	*P*-value
**Overall survival**				
T Stage (T3-T4)	5.68 (2.44–13.22)	**< 0.001**	5.21 (2.20–12.37)	**< 0.001**
Angiolymphatic invasion (present)	4.21 (1.85–9.61)	**0.001**	3.72 (1.60–8.65)	**0.002**
N stage (N2)	2.37 (0.93–6.02)	0.070		
Age (≥55)	1.86 (0.73–4.73)	0.194		
Gender (female)	1.61 (0.69–3.75)	0.270		
Perineural invasion (present)	1.55 (0.65–3.66)	0.321		
PD-1+ cell number (high; continuous variable)	1.01 (0.95–1.06))	0.826		
miR-197 expression (high; continuous variable)	1.01 (1.00–1.02)	**0.033**		NS
CD8+ cell number (high; continuous variable)	0.99 (0.97–1.01)	0.181		
CD3+ cell number (high; continuous variable)	0.99 (0.98–1.00)	0.142		
CD4+ cell number (high; continuous variable)	0.98 (0.96–1.00)	0.082		
CD20+ cell number (high; continuous variable)	0.98 (0.96–1.00)	0.186		
FoxP3+ cell number (high; continuous variable)	0.98 (0.93–1.03)	0.374		
PD-L1 expression (2+)	0.32 (0.11–0.94)	**0.039**		NS
**Disease-free survival**				
T Stage (T3-T4)	7.74 (2.67–22.42)	**< 0.001**	6.69 (2.25–19.84)	**0.001**
Angiolymphatic invasion (present)	5.88 (2.13–16.27)	**0.001**	4.90 (1.72–13.95)	**0.003**
N stage (N2)	4.04 (1.46–11.15)	**0.007**		NS
Perineural invasion (present)	2.35 (0.87–6.31)	0.092		
Gender (female)	1.68 (0.63–4.52)	0.303		
FoxP3+ cell number (high; continuous variable)	1.01 (0.96–1.06)	0.754		
miR-197 expression (high; continuous variable)	1.01 (1.00–1.02)	0.089		
CD3+ cell number (high; continuous variable)	1.00 (0.98–1.01)	0.552		
CD20+ cell number (high; continuous variable)	0.99 (0.97–1.02)	0.496		
CD8+ cell number (high; continuous variable)	0.99 (0.96–1.01)	0.282		
CD4+ cell number (high; continuous variable)	0.98 (0.96–1.01)	0.187		
PD-1+ cell number (high; continuous variable)	0.94 (0.85–1.04))	0.213		
Age (≥55)	0.79 (0.30–2.13)	0.646		
PD-L1 expression (2+)	0.25 (0.06–1.12)	0.070		

HR, harzard ratio; CI, confidence interval; NS, not significant.

### Survival analysis in miR-197^low^ and miR-197^high^ subgroups

Because our results suggested that miR-197 inhibited PD-L1 expression in OSCC, we investigated correlations of PD-L1 with survival according to miR-197 level (Table 5 and Figure 3). In the miR-197^high^ subgroup (>median value of miR-197; n=34), prognostic factors for OS in univariate analysis, namely, PD-L1, T stage (T3-T4), and angiolymphatic invasion, were incorporated into the multivariate analysis, resulting in PD-L1 and angiolymphatic invasion as independent prognostic factors for OS (*P* = 0.040 and *P* = 0.035), whereas angiolymphatic invasion was the only prognostic factor for DFS (*P* = 0.009). In contrast, multivariate analysis of the miR-197^low^ subgroup (<median value of miR-197; n = 34) showed that T stage (T3-T4) and angiolymphatic invasion were independently associated with OS (*P* = 0.005 and *P* = 0.040), and T stage (T3-T4) was an independent prognostic factor for DFS (*P* = 0.001), with no prognostic significance of PD-L1 for OS or DFS in the miR-197^low^ subgroup. Therefore, high PD-L1 expression (2+) was a favorable prognostic factor for OS only in the miR-197^high^ subgroup, suggesting that prognostic implication of PD-L1 is influenced by the miR-197 level.

**Table 5:** Prognostic factors for overall survival and disease-free survival in miR-197^high^ (n = 34) and miR-197^low^ (n = 34) subgroups of oral squamous cell carcinoma

miR-197^high^ subgroup (miR-197 > median)				
Variables	Univariate analysis^†^		Multivariate analysis^†^	
	HR (95% CI)	*P*-value	HR (95% CI)	*P*-value
**Overall survival**				
Angiolymphatic invasion (present)	3.31 (1.16–9.45)	**0.025**	3.16 (1.09–9.17)	**0.035**
N stage (N2)	3.17 (0.86–11.69)	0.084		
T Stage (T3-T4)	2.85 (1.02–7.96)	**0.045**		NS
Age (≥55)	2.13 (0.60–7.57)	0.243		
Gender (female)	1.43 (0.48–4.24)	0.523		
FoxP3+ cell number (high; continuous variable)	1.00 (0.95–1.05)	0.897		
CD8+ cell number (high; continuous variable)	0.99 (0.97–1.01)	0.463		
CD3+ cell number (high; continuous variable)	0.99 (0.98–1.01)	0.244		
CD4+ cell number (high; continuous variable)	0.99 (0.97–1.01)	0.366		
CD20+ cell number (high; continuous variable)	0.98 (0.95–1.01)	0.244		
PD-1+ cell number (high; continuous variable)	0.98 (0.90–1.06)	0.599		
Perineural invasion (present)	0.64 (0.18–2.25)	0.482		
PD-L1 expression (2+)	0.28 (0.09–0.90)	**0.033**	0.29 (0.09–0.95)	**0.040**
**Disease-free survival**				
Angiolymphatic invasion (present)	6.92 (1.64–29.33)	**0.009**	6.92 (1.64–29.33)	**0.009**
N stage (N2)	6.07 (1.42–25.86)	**0.015**		NS
T Stage (T3-T4)	4.15 (0.99–17.40)	0.052		
Perineural invasion (present)	1.57 (0.38–6.59)	0.535		
Gender (female)	1.49 (0.36–6.23)	0.587		
Age (≥55)	1.47 (0.30–7.29)	0.637		
FoxP3+ cell number (high; continuous variable)	1.03 (0.96–1.10)	0.412		
CD3+ cell number (high; continuous variable)	0.99 (0.97–1.01)	0.418		
CD8+ cell number (high; continuous variable)	0.98 (0.95–1.02)	0.307		
CD20+ cell number (high; continuous variable)	0.98 (0.94–1.02)	0.325		
CD4+ cell number (high; continuous variable)	0.98 (0.94–1.01)	0.221		
PD-1+ cell number (high; continuous variable)	0.96 (0.85–1.08)	0.503		
PD-L1 expression (2+)	0.13 (0.02–1.06)	0.057		
**Overall survival**				
T Stage (T3-T4)	15.90 (3.13–80.85)	**0.001**	11.80 (2.13–65.44)	**0.005**
Angiolymphatic invasion (present)	9.25 (1.85–46.33)	**0.007**	6.21 (1.09–35.47)	**0.040**
Perineural invasion (present)	5.64 (1.33–23.95)	**0.019**		NS
N stage (N2)	2.75 (0.66–11.54)	0.166		
Gender (female)	1.98 (0.49–7.96)	0.334		
Age (≥55)	1.36 (0.32–5.68)	0.678		
PD-1+ cell number (high; continuous variable)	1.02 (0.94–1.10)	0.675		
CD20+ cell number (high; continuous variable)	0.98 (0.94–1.02)	0.382		
CD3+ cell number (high; continuous variable)	0.97 (0.93–1.01)	0.135		
CD4+ cell number (high; continuous variable)	0.97 (0.93–1.01)	0.129		
CD8+ cell number (high; continuous variable)	0.94 (0.88–1.00)	0.054		
FoxP3+ cell number (high; continuous variable)	0.85 (0.70–1.03)	0.101		
PD-L1 expression (2+)	0.04 (0.00–59.97)	0.378		
**Disease-free survival**				
T Stage (T3-T4)	15.29 (2.99–78.18)	**0.001**	15.29 (2.99–78.18)	**0.001**
Angiolymphatic invasion (present)	4.88 (1.16–20.55)	**0.031**		NS
Perineural invasion (present)	3.15 (0.78–12.76)	0.107		
N stage (N2)	2.92 (0.70–12.27)	0.144		
Gender (female)	1.88 (0.47–7.52)	0.374		
CD3+ cell number (high; continuous variable)	1.00 (0.98–1.03)	0.800		
CD20+ cell number (high; continuous variable)	1.00 (0.97–1.03)	0.913		
CD4+ cell number (high; continuous variable)	0.99 (0.96–1.02)	0.540		
CD8+ cell number (high; continuous variable)	0.99 (0.95–1.04)	0.741		
FoxP3+ cell number (high; continuous variable)	0.98 (0.87–1.09)	0.660		
PD-1+ cell number (high; continuous variable)	0.84 (0.64–1.10)	0.197		
Age (≥55)	0.47 (0.11–1.98)	0.304		
PD-L1 expression (2+)	0.59 (0.07–4.79)	0.621		

## DISCUSSION

In the tumor microenvironment, the PD-L1/PD-1 axis has become the therapeutic locus in various cancers. In this study, we focused on the clinicopathologic implication of the miR-197/PD-L1 axis and the profiles of recruited TILs in OSCC. We observed the inverse correlation between miR-197 and PD-L1, and the positive correlation between PD-L1 and TILS, suggesting an active network of miR-197/PD-L1/TILs. Aggressive clinicopathologic features were associated with depleted TILs, and high T stage (T4) disease was associated with low PD-L1 but high miR-197 expression. Moreover, high PD-L1 expression (2+) was an independent favorable prognostic factor for overall survival (OS) (*P* = 0.040) in the miR-197^high^ subgroup, but not in the miR-197^low^ subgroup.

MiR-197 is upregulated in lung, liver, and thyroid cancers and targets a variety of genes promoting cell proliferation and inhibiting apoptosis [11, 22, 23]. MiR-197 has an indirect effect on PD-L1, which has already been investigated in a study of NSCLC [12]. In the NSCLC study, the miR-197/CKS1B/STAT3-induced PD-L1 network leads to tumor progression, and miR-197 is an inverse indicator of PD-L1 expression and predicts shorter OS [12]. Consistent with this result, we observed that miR-197 level inversely correlated with PD-L1 expression detected by immunohistochemistry in OSCC, suggesting that miR-197, at least in part, suppresses PD-L1 expression in OSCC. The proposed mechanism in NSCLC explains that miR-197 has a direct inhibitory effect on the cyclin-dependent kinase CKS1B, which promotes PD-L1 expression by activating STAT3 [12]. Although the detailed mechanism of PD-L1 expression remains to be clarified, our observations suggest that the putative miR-197-mediated negative regulation of PD-L1 might also have clinical significance in OSCC.

The mechanisms of PD-L1 expression in tumor cells have been suggested to include two major pathways. The extrinsic signaling pathway is activated by interferon-γ (IFN-γ) produced by TILs, resulting in JAK/STAT activation in tumor cells, and the intrinsic pathway is activated by PI3K/AKT/mTOR signaling [24, 25]. In this context, miR-197 possibly suppress the downstream signaling of the extrinsic pathway of PD-L1 expression via CKS1B/STAT3 pathway, and therefore, the resultant PD-L1 expression level might reflect the signals from IFN-γ-producing T cells modified by miR-197, as well as signals from the intrinsic pathway. Considering the inverse correlation of miR-197 and PD-L1 in our study, PD-L1 expression in OSCC might be dependent on the miR-197-mediated negative regulation, which could provide the different prognostic significance of PD-L1 according to the miR-197 level.

We divided our OSCC cohort into miR-197^high^ and miR-197^low^ subsets, reflecting the mechanism of PD-L1 expression. PD-L1 had prognostic significance only in the miR-197^high^ subset, although it is not yet clear how high expression of miR-197 influences prognostic significance of PD-L1 in addition to expression of PD-L1. It is possible that OSCC with high miR-197 expression at a sufficient level might have an inhibitory effect on the signal from the extrinsic pathway induced by STAT3, while sparing the intrinsic signal. This skewing effect of the PD-L1 pathway with co-activated Akt/mTOR-related downstream signals could influence the biology of OSCC in a complex manner, which remains to be clarified further.

Attempts have been made to identify the relation between PD-L1 expression and the effectiveness of immune-induced host defense against the tumor measured by TILs and prognosis of patients [26-28]. In this study, we observed that the higher density of TILs was associated with high PD-L1 expression in tumor cells of OSCC. Consistent with our results, previous studies reported the correlation between PD-L1 expression of tumor and microenvironmental TILs in cancers of the colon, breast, larynx, and thymus [28-31]. Moreover, studies that detected the PD-L1 mRNA expression showed that high PD-L1 expression is associated with increased TILs and favorable prognosis, as shown in our study [29-32]. These results seem paradoxical, given the inhibitory role of PD-L1 in immune responses. Considering the conflicting results of the PD-L1 association with TILs and prognosis according to tumor types or even within the same type of tumors [27, 33, 34], PD-L1 expression of tumor or recruited number of TILs might not be a surrogate marker for the final status of effective immune evasion, but a marker of active immune response by tumor cells to evade anti-tumor immune attack by host TILs, regardless of its effectiveness. Therefore, high PD-L1 expression and accumulated TILs in our study might reflect unsuccessfully skewed immune evasion [35], which remains to be clarified further.

In the present study of OSCC, high miR-197 was associated with higher tumor stage (T4) and poor prognosis, whereas high PD-L1 expression in tumors was associated with good prognosis. Although TILs did not directly correlate with survival of OSCC patients, low numbers of various TILs were associated with aggressive features of tumors, including high stage, angiolymphatic invasion, and perineural invasion. The phase of anti-tumor immune reaction might be different between early tumors and full-blown tumors because of different exposure time and amount of tumor antigens, and the mechanism of associations between miR-197/PD-L1/TILs and stages of disease must be investigated further. Considering that many clinicopathologic factors and outcomes can influence or be influenced by anti-tumor immune reactions [36], comprehensive analyses of miR-197/PD-L1 and TILs could be more useful than individual analysis of PD-L1. Studies suggest that the miRNA is a factor in controlling the PD-1/PD-L1 signaling for cancer immunotherapy [18, 37]. Our approach with miR-197, PD-L1, and TILs could be effective in recognizing the clinicopathologic implication of miR-197/PD-L1 and TILs, and selecting patients for immunotherapeutic strategy and avoiding immune-related adverse reactions in OSCC.

Studies related to the expression of PD-L1 and its prognostic effects in different cancer types show discordant results [38-40]. Various clinicopathologic factors, including cancer types and individual host factors could influence pattern, degree, and effectiveness of anti-tumor immune reactions. Furthermore, different methods of detecting PD-L1 protein or mRNA have been developed, including IHC that utilizes various antibodies [41], in situ hybridization assay [32], DNA microarrays [29], and quantitative real-time PCR [30, 31]. In addition, pre-operative neoadjuvant treatment might influence expression of PD-L1, as well as miR-197 and TILs. In this study, neoadjuvant treatment was administered in only 4.4% of patients, with no significant correlation with clinicopathologic variables, suggesting limited effects of neoadjuvant treatments. Because these various factors and techniques might make the results of PD-L1 expression widely variable, previous clinicopathologic studies must be interpreted carefully [42]. In this context, our miR-197/PD-L1-paired approach can contribute to understanding the mechanism and clinical significance of PD-L1–associated anti-tumor host immune function in OSCC.

This study showed inverse correlation and prognostic effects between miR-197 and PD-L1 expression in OSCC. In addition, we observed that PD-L1 expression on IHC is associated with increased TILs and favorable prognosis in miR-197^high^ subgroup. Our clinicopathologic analysis of miR-197/PD-L1 and TILs could contribute to understanding of clinical significance of anti-tumor immune reactions in OSCC.

## MATERIALS AND METHODS

### Patients, samples, clinical data, and study design

A total of 68 OSCC patients who underwent curative surgery between 2003 and 2011 at Seoul National University Bundang Hospital were enrolled. We reviewed hematoxylin-eosin (H&E), immunohistochemically stained slides, pathology reports, and medical records to establish the clinicopathologic features of the tumors. TNM staging was determined according to the seventh edition of the American Joint Committee on Cancer guidelines [43]. Tissue microarrays were constructed by collecting single-tissue cores (2 mm in diameter) from the most morphologically representative areas of formalin-fixed, paraffin-embedded (FFPE) tissue specimens, as previously described [44]. In the analysis of the clinical data, disease-free survival (DFS) was defined as survival time from curative surgical resection to the last follow-up date of disease-free state or to death. Overall survival (OS) was defined as survival time from curative surgical resection to death by any cause. The time intervals for the regular follow-up was 3 months after surgery for the first 2 years, and then every 6 months for the next 3 years. The follow-up period changed if the patient had symptoms or signs that possibly correlated with disease or treatment at the regular visit. Mean follow-up duration was 44.3 months (range, 2.1-122.0 months). During the follow-up period, 28% (19/68) of patients had recurrence and 34% (23/68) of patients died. The remainder of the patients were considered censored in survival analysis for DFS and OS. The primary objective was to determine the correlation among miR-197, PD-L1, TILs, and clinicopathologic factors. The secondary objective was to determine the prognostic effects of miR-197, PD-L1, and TILs on OS and DFS.

### RNA extraction and quantitative PCR for miR-197 expression analysis

Total RNA was extracted from 10-μm-thick FFPE tissue sections by use of a RecoverAll Total Nucleic Acid Isolation Kit for FFPE samples (Applied Biosystems, Foster City, CA, USA), and stored at −80° C until the time of use after measuring the concentration with a NanoDrop 2000 spectrophotometer (Thermo Fisher Scientific, Waltham, MA, USA). To measure the relative expression level of hsa-miR-197 (Catalog no. #4427975, Applied Biosystems), reverse transcription and real-time polymerase chain reaction were conducted by use of 10 ng of total RNA, Universal PCR Master Mix and a TaqMan microRNA Reverse Transcription kit (Catalog no. 4366596, Applied Biosystems) with U6 snRNA as an internal reference gene and reactive tonsil tissue as a normal control, as previously described [45]. The relative level of miR-197 in OSCC was calculated as 2^-ΔΔCt^, where ΔCt = Ct (miR-197) – Ct (U6) and ΔΔCt = ΔCt(tumor) – ΔCt (normal).

### Immunohistochemistry

Immunohistochemistry (IHC) staining was applied to TMA sections (4 μm) by use of the Ventana Benchmark XT automated staining system (Ventana Medical Systems, Tucson, AZ, USA). The following primary antibodies were used: polyclonal rabbit anti-human PD-L1 (clone ab153991, Abcam, Cambridge, UK; 1:1000), monoclonal rabbit anti-human PD-1 (clone #6796-1, Epitomics, Burlingame, CA, USA; 1:300), monoclonal rabbit anti-human CD4 (clone SP35, Catalog no. 790-4423, Ventana Medical Systems), monoclonal mouse anti-human CD8 (clone C8/144B, Catalog no. IR623, Dako, Carpinteria, CA, USA), mouse monoclonal anti-human FoxP3 (clone #236A/E7, Catalog no. ab20034, Abcam; 1:50), rabbit monoclonal anti-human CD3 (clone #SP7, Catalog no. RM-9107-S, Thermo Scientific, Waltham, MA, USA; 1:100), mouse monoclonal anti-human CD20 (clone #L26, Catalog no. IR604, Dako). Chorionic villi of human placenta were used as positive control, and human skin tissue was used as negative control. In this setting, we adjusted the staining condition for human oral cancer tissues. Immunostaining was interpreted by two pathologists (H.A. and J.H.P.). PD-L1 was stained in cytoplasm or cell membrane of tumor cells or both and graded as 0, 1+ and 2+, according to the intensity of PD-L1.

### Interpretation of immunohistochemical staining

All immunostained slides were blindly evaluated by two experienced pathologists (H.A. and J.H.P.). PD-L1 IHC was defined as membranous or by cytoplasmic positivity and scored as follows: 0 indicates no staining or staining in <10% of the tumor cells, 1+ indicates staining in ≥10% of the tumor cells with weak positivity, and 2+ indicates moderate to strong positivity, which was modified from several previous studies [46-48]. The numbers of CD3+, CD4+, CD8+, PD-1+, FoxP3+, and CD20+ TILs around the tumor cells were counted in three representative areas under a high-power optical microscope (400x). The average absolute number was recorded.

### Statistical analysis

Statistical analysis was done mainly by SPSS Statistic Version 21.0 software package (IBM Corp., Armonk, NY, USA). Chi-square tests, Student’s *t*-test, Pearson’s correlation test, and Spearman’s rho test were used to examine the relations among expression of miR-197, PD-L1, number of TILs, and clinicopathologic variables. Survival analysis was performed by use of the Cox regression hazard model. For the multivariate survival analysis, clinicopathologic variables with significant *P*-values (<0.05) in univariate analysis were entered into the multivariate Cox model, and *P*-values of <0.05 were considered significant. R packages (Version 3.1.2) (http://www.r-project.org) were used in the graphical dot-plot analysis [49-51].

## SUPPLEMENTARY MATERIALS TABLES






