# Supplementary material for: Clinicopathologic implications of the miR-197/PD-L1 axis in oral squamous cell carcinoma
Source: Oncotarget. 2017 Aug 3;8(39):66178–94. doi: 10.18632/oncotarget.19842 (PMC5630402; doi:10.18632/oncotarget.19842)
Supplement: Supplementary file 1 [file oncotarget-08-66178-s001.pdf]

Clinicopathologic implications of the miR-197/PD-L1 axis in oral squamous cell carcinoma

SUPPLEMENTARY MATERIALS

Supplementary Table 1: Clinicopathologic features of oral squamous cell carcinoma according to miR-197 expression level (n=68).

See Supplementary File 1

Supplementary Table 2: Clinicopathologic features of oral squamous cell carcinoma according to tumor expression of PD-L1 (n=68).

See Supplementary File 2
