# Supplementary material for: Clinicopathologic implications of the miR-197/PD-L1 axis in oral squamous cell carcinoma
Source: Oncotarget. 2017 Aug 3;8(39):66178–94. doi: 10.18632/oncotarget.19842 (PMC5630402; doi:10.18632/oncotarget.19842)
Supplement: Supplementary file 2 [file oncotarget-08-66178-s002.docx]

**Supplementary Table 1: Clinicopathologic features of oral squamous cell carcinoma according to miR-197 expression level (n=68)**

| Clinicopathologic variables | Number of patients  (total n=68) | miR197-  Low (n=34; <median) |  | miR197-High (n=34; >median) |  | *p*-value |
| --- | --- | --- | --- | --- | --- | --- |
|  |  | N | % | N | % |  |
| Age |  |  |  |  |  | 0.209 |
| <55 | 25 | 15 | 60.0% | 10 | 40.0% |  |
| ≥55 | 43 | 19 | 44.2% | 24 | 55.8% |  |
| Gender |  |  |  |  |  | 0.442 |
| Male | 45 | 21 | 46.7% | 24 | 53.3% |  |
| Female | 23 | 13 | 56.5% | 10 | 43.5% |  |
| AJCC Stage |  |  |  |  |  | 0.544 |
| Stage 1-2 | 35 | 16 | 45.7% | 19 | 54.3%. |  |
| Stage 3-4 | 33 | 18 | 54.5% | 15 | 45.5% |  |
| AJCC Tumor Stage |  |  |  |  |  | 0.431 |
| pT1-2 | 47 | 25 | 53.2% | 22 | 46.8% |  |
| pT3-4 | 21 | 9 | 42.9% | 12 | 57.1% |  |
| AJCC Lymph node Stage |  |  |  |  |  | 0.134 |
| pN0 | 42 | 18 | 42.9% | 24 | 57.1% |  |
| pN1-2 | 26 | 16 | 61.5% | 10 | 38.5% |  |
| Angiolymphatic invasion |  |  |  |  |  | 0.417 |
| Not identified | 49 | 23 | 46.9% | 26 | 53.1% |  |
| Present | 19 | 11 | 57.9% | 8 | 42.1% |  |
| Perineural invasion |  |  |  |  |  | 0.787 |
| Not identified | 49 | 24 | 49.0% | 25 | 51.0% |  |
| Present | 19 | 10 | 52.6% | 9 | 47.4% |  |
| Survival |  |  |  |  |  | 0.073 |
| Survival | 45 | 26 | 57.8% | 19 | 42.2% |  |
| Death | 23 | 8 | 34.8% | 15 | 65.2% |  |
| Relapse |  |  |  |  |  | 0.417 |
| No relapse | 49 | 26 | 53.1% | 23 | 46.9% |  |
| Relapse | 19 | 8 | 42.1% | 11 | 57.9% |  |
| Neoadjuvant  treatment |  |  |  |  |  | 0.555 |
| Not done | 65 | 32 | 49.2% | 33 | 50.8% |  |
| Done | 3 | 2 | 66.7% | 1 | 33.3% |  |
| Additional  treatment |  |  |  |  |  | 0.808 |
| Not done | 31 | 15 | 48.4% | 16 | 51.6% |  |
| Done | 37 | 19 | 51.4% | 18 | 48.6% |  |
