# Supplementary material for: Clinicopathologic implications of the miR-197/PD-L1 axis in oral squamous cell carcinoma
Source: Oncotarget. 2017 Aug 3;8(39):66178–94. doi: 10.18632/oncotarget.19842 (PMC5630402; doi:10.18632/oncotarget.19842)
Supplement: Supplementary file 3 [file oncotarget-08-66178-s003.docx]

**Supplementary Table 2: Clinicopathologic features of oral squamous cell carcinoma according to tumor expression of PD-L1 (n=68)**

| Clinicopathologic variables | Number of patients  (total n=68) | PDL1-Low (0/1) |  | PDL1-High (2) |  | *p*-value |
| --- | --- | --- | --- | --- | --- | --- |
|  |  | N | % | N | % |  |
| Age |  |  |  |  |  | 0.624 |
| <55 | 25 | 16 | 64.0% | 9 | 36.0% |  |
| ≥55 | 43 | 30 | 69.8% | 13 | 30.2% |  |
| Gender |  |  |  |  |  | 0.430 |
| Male | 45 | 29 | 64.4% | 16 | 35.6% |  |
| Female | 23 | 17 | 73.9% | 6 | 26.1% |  |
| AJCC Stage |  |  |  |  |  | 0.385 |
| Stage 1-2 | 35 | 22 | 62.9% | 13 | 37.1%. |  |
| Stage 3-4 | 33 | 24 | 72.7% | 9 | 27.3% |  |
| AJCC Tumor Stage |  |  |  |  |  | 0.314 |
| pT1-2 | 47 | 30 | 63.8% | 17 | 36.2% |  |
| pT3-4 | 21 | 16 | 76.2% | 5 | 23.8% |  |
| AJCC Lymph node Stage |  |  |  |  |  | 0.451 |
| pN0 | 42 | 27 | 64.3% | 15 | 35.7% |  |
| pN1-2 | 26 | 19 | 73.1% | 7 | 26.9% |  |
| Angiolymphatic invasion |  |  |  |  |  | 0.215 |
| Not identified | 49 | 31 | 63.3% | 18 | 36.7% |  |
| Present | 19 | 15 | 78.9% | 4 | 21.1% |  |
| Perineural invasion |  |  |  |  |  | 0.932 |
| Not identified | 49 | 33 | 67.3% | 16 | 32.7% |  |
| Present | 19 | 13 | 68.4% | 6 | 31.6% |  |
| Survival |  |  |  |  |  | 0.059 |
| Alive | 45 | 27 | 60.0% | 18 | 40.0% |  |
| Death | 23 | 19 | 82.6% | 4 | 17.4% |  |
| Relapse |  |  |  |  |  | 0.069 |
| No relapse | 49 | 30 | 61.2% | 19 | 38.8% |  |
| Relapse | 19 | 16 | 84.2% | 3 | 15.8% |  |
| Neoadjuvant  treatment |  |  |  |  |  | 0.221 |
| Not done | 65 | 43 | 66.2% | 22 | 33.8% |  |
| Done | 3 | 3 | 100.0% | 0 | 0.0% |  |
| Additional  treatment |  |  |  |  |  | 0.305 |
| Not done | 31 | 19 | 61.3% | 12 | 38.7% |  |
| Done | 37 | 27 | 73.0% | 10 | 27.0% |  |
